# OLDER ADULTS’ OPINIONS ON DIFFERENT VEHICLE TECHNOLOGIES

**DOI:** 10.1093/geroni/igz038.1241

**Published:** 2019-11-08

**Authors:** Sara A Freed, Lesley A Ross, Despina Stavrinos

**Affiliations:** 1 The Pennsylvania State University, University Park, Pennsylvania, United States; 2 The University of Alabama at Birmingham, Birmingham, Alabama, United States

## Abstract

Vehicle technologies have the potential to greatly improve road safety. Given normative changes in cognitive, sensory and physical functioning, older drivers may particularly benefit from such technologies. However, little research has examined older adults’ opinions of vehicle technologies, descriptive differences of individuals more likely to have positive opinions of vehicle technologies, and how their opinions may differ depending on the type of vehicle technology. The current study examined older adults’ opinions on vehicle technology in a sample of 72 adults between 65 and 85 years (M = 72.3, SD = 5.36, 48% women). Participants were asked, “How important is [parking assistance, crash avoidance systems, early collision warnings, built-in GPS] in choosing a new vehicle?” on a scale from 1 (“not at all”) to 5 (“must have”). On average, participants rated built-in GPS as the most important (M = 3.81, SD=1.10) with parking assist as the least important (M=1.86, SD=1.13). We used correlational analyses to examine the association between demographic and personality and importance ratings. Women were more likely to rate greater importance for built-in GPS than men (r=.35, p<.05). Age, education, self-reported driving quality, and self-reported average weekly driving miles were not significantly associated with importance rating. In terms of personality, only higher levels of extraversion were associated with more positive ratings of early collision warning systems (r=.24, p<.05). Vehicle technology design and education should take older adults’ preferences into account and consider individual differences, and future work should examine other predictors of vehicle technology preferences such as functional performance.

